# Multiple Pyogenic Liver Abscesses Mimicking Metastatic Disease on Computed Tomography

**DOI:** 10.7759/cureus.7050

**Published:** 2020-02-19

**Authors:** Esra Özgül

**Affiliations:** 1 Radiology, Afyonkarahisar Health Science University, Afyonkarahisar, TUR

**Keywords:** liver abscesses, metastasis, abdomen, computed tomography, pyogenic

## Abstract

Liver abscesses are divided into two main subgroups: pyogenic and nonpyogenic abscesses. Early diagnosis is important for appropriate treatment and to reduce the morbidity and mortality rates in liver abscesses. We report a case of multiple pyogenic liver abscesses mimicking liver metastases on multidetector computed tomography (MDCT). The case is unique as it shows a rare presentation of pyogenic liver abscess that cannot be distinguished from metastatic liver disease. Microbiologic and pathologic correlations with follow-up may be necessary for these patients. The case is presented with an emphasis on the MDCT findings.

## Introduction

Early diagnosis of a hepatic abscess is important to reduce its morbidity and mortality [1]. In spite of appropriate treatment, the mortality rate is still about 30% and ranges from 11% to 88% in the literature [2]. Liver abscesses are divided into two subgroups: pyogenic and nonpyogenic. Early diagnosis is important for appropriate treatment and to reduce the morbidity and mortality rates in liver abscesses. Imaging features such as scintigraphy, ultrasound, and multidetector computed tomography (MDCT) are important for the diagnosis. Computed tomography (CT), with high sensitivity, is the most accurate method of detecting liver abscesses [3]. Generally on contrast-enhanced CT scans, pyogenic liver abscesses reveal a centrally non-enhancing abscess cavity with an enhancing capsule. Usually, with this contrast enhancement pattern, it is easy to differentiate metastases from a liver abscess. Here, we report the appearance of multiple, widely scattered pyogenic liver abscesses that mimicked liver metastases in a diabetic woman. In this case, it was difficult to distinguish the abscesses from the metastatic lesions with the MDCT findings. Therefore, microbiologic and pathologic correlation with follow-up may be necessary in these patients.

## Case presentation

A 57-year-old woman was admitted to our emergency department with complaints of right upper quadrant abdominal pain and nausea. On admission, her body temperature was 38.6ºC; her pulse rate and blood pressure were within normal limits. On physical examination, mild tenderness was found over the right upper quadrant. There was mild hepatomegaly on palpation, and her scleras were icteric. There was no rebound tenderness. Bowel sounds were normal, and there was no splenomegaly. Laboratory studies revealed leukocytosis (17.1 × 10^3^/µL (normal range, 4.5-11 × 10^3^/µL), elevated C-reactive protein at 315.8 mg/L (normal range, 0-10 mg/L), and elevation of liver enzymes. Results of the other laboratory tests, including pancreatic enzymes, electrolytes, and urinalyses, were within normal limits. Blood cultures were obtained from the patient.

An abdominal ultrasound was performed, and multiple hypoechoic round-shaped lesions were seen within the liver parenchyma. No vascularity was obtained from the lesions with color and power Doppler ultrasound examination. However, most of the lesions were very small (less than 2.5 cm) and scattered everywhere within the liver parenchyma. The lesions were thought to be metastatic disease, so the patient had an abdominal MDCT examination performed to characterize the lesions further and to discover the primary focus. Abdominal MDCT with oral and intravenous contrast was performed. Precontrast 5-mm-thick sections were taken after oral contrast administration. The examination was repeated after 150 mL of noniodinated IV-contrast agent administration on the portal and late venous phases. Thoracic MDCT examination also was performed during the arterial phase.

**Figure 1:**
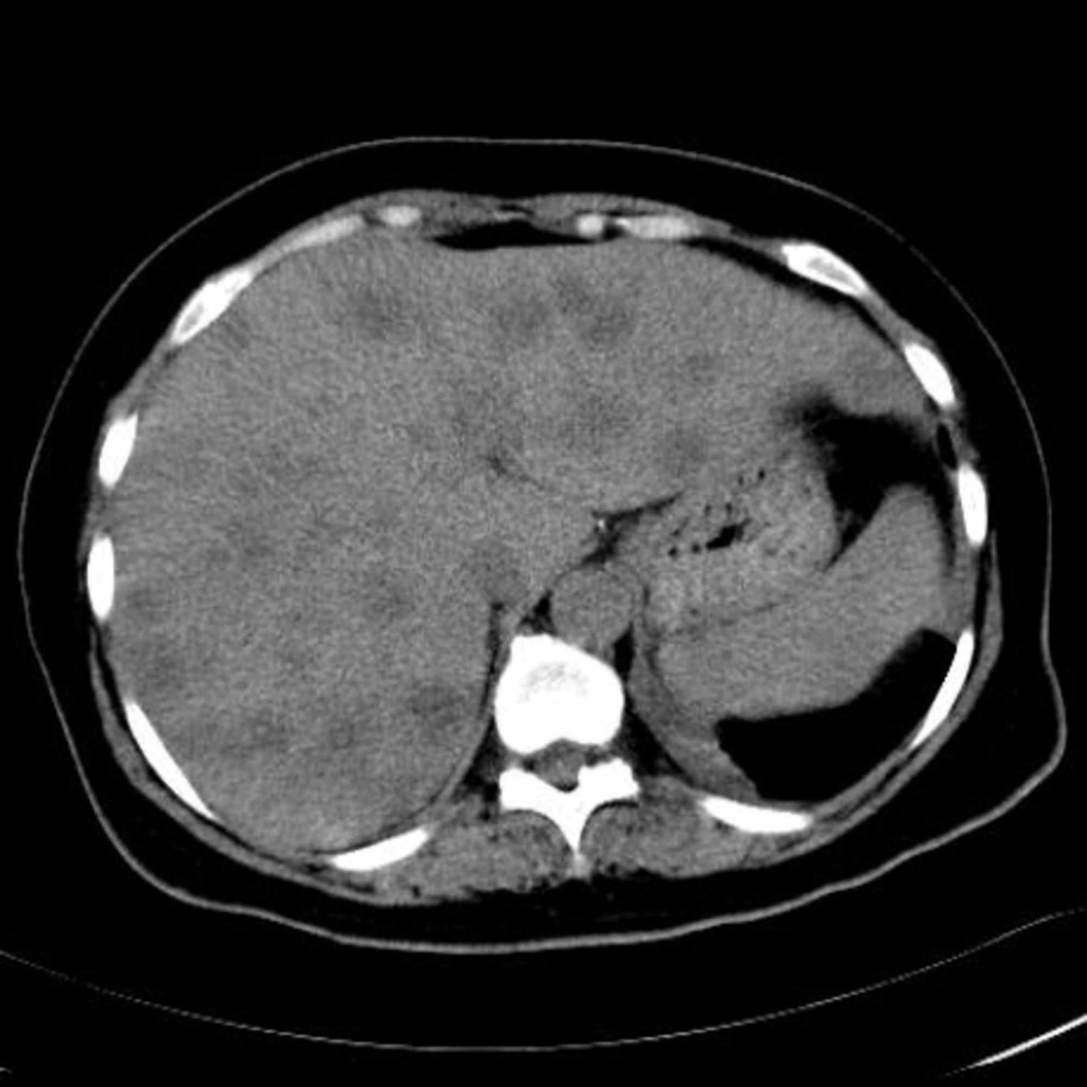
Abdominal precontrast MDCT image demonstrates multiple hypodense lesions scattered throughout the liver parenchyma.

**Figure 2:**
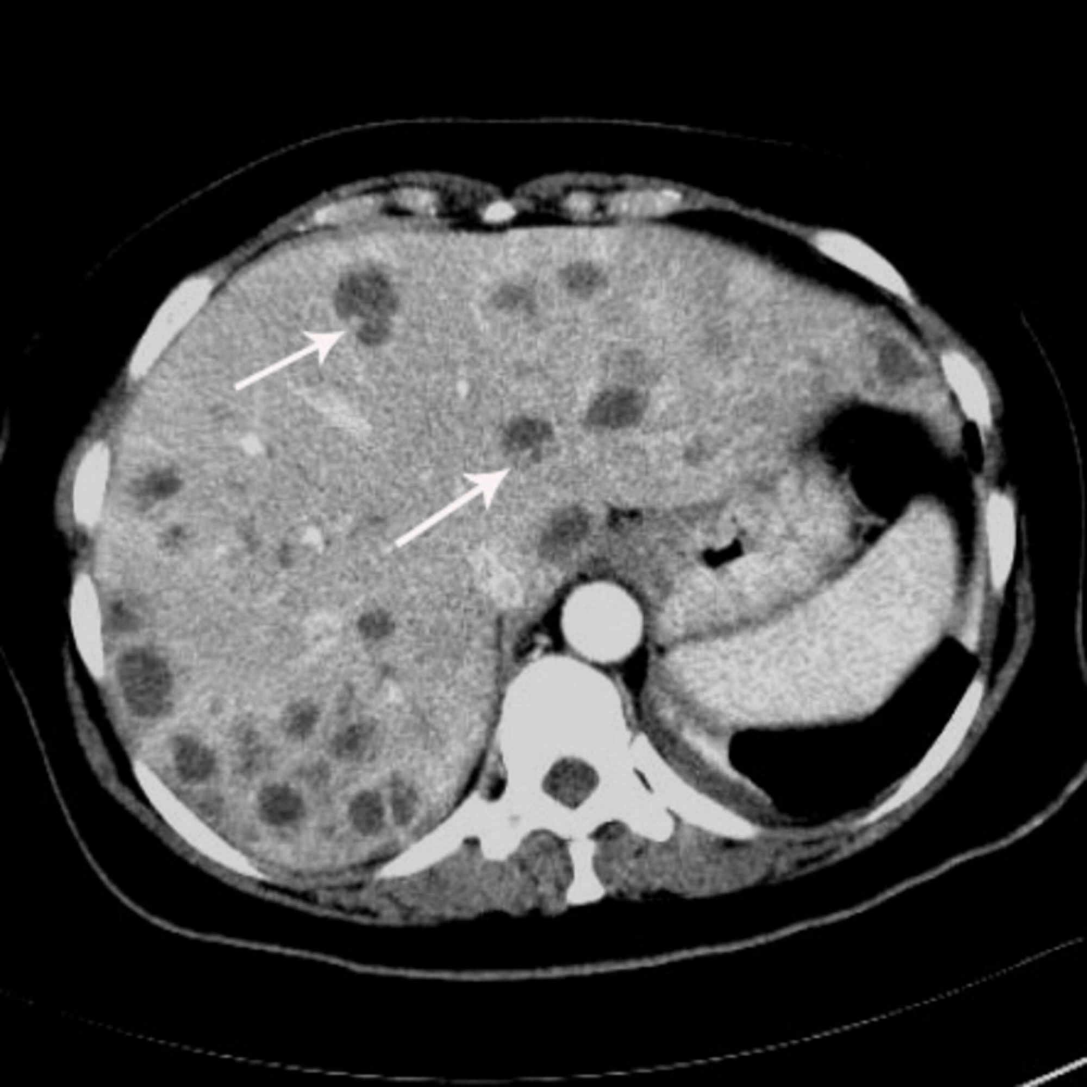
In the portal phase image, the lesions show peripheral heterogeneous enhancement (arrows).

**Figure 3:**
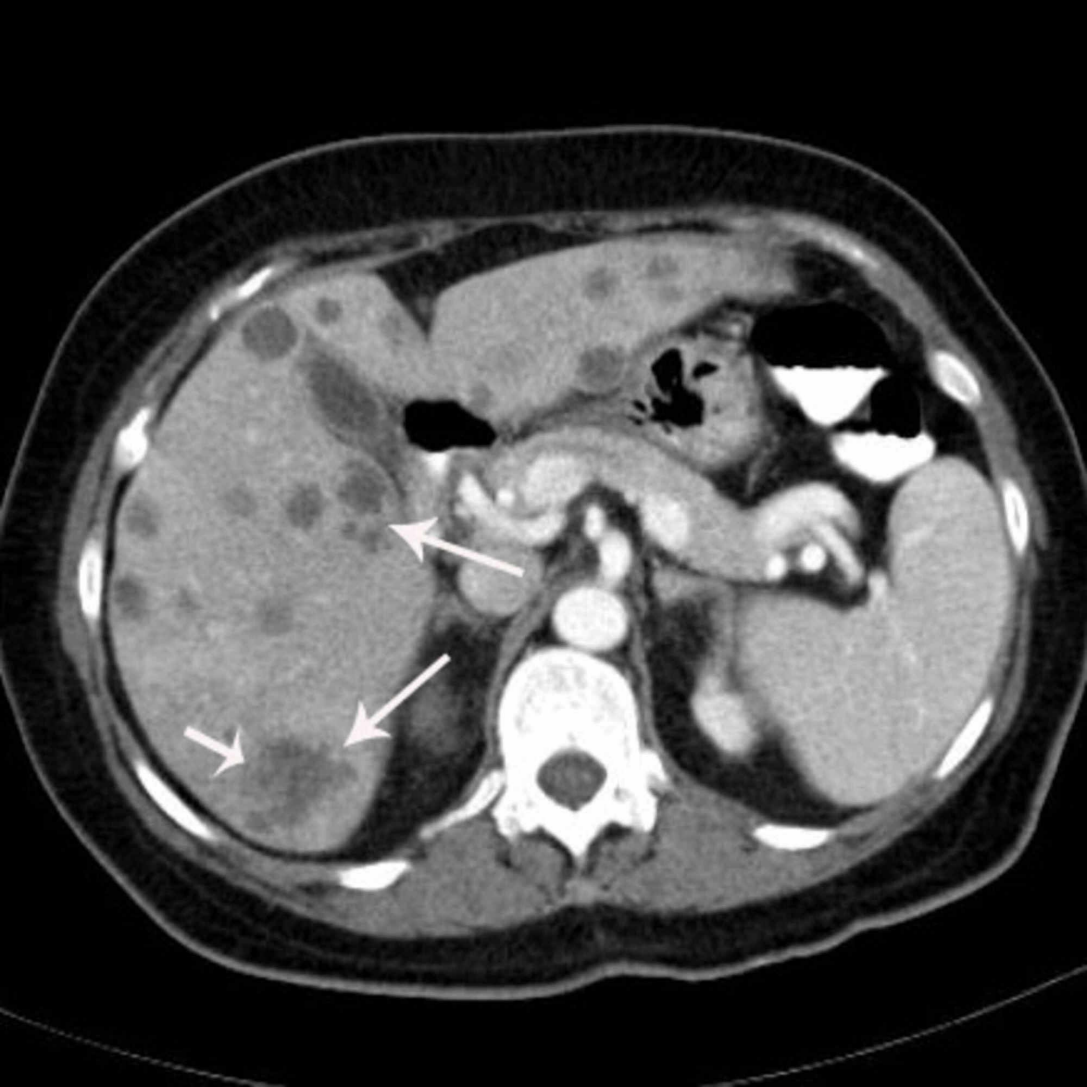
A portal phase image of the liver from a different level reveals lesions showing peripheral heterogeneous enhancement and contrast enhancement in some of the lesions (long white arrows). The largest lesion measures 2.5 cm in diameter (short white arrow).

**Figure 4:**
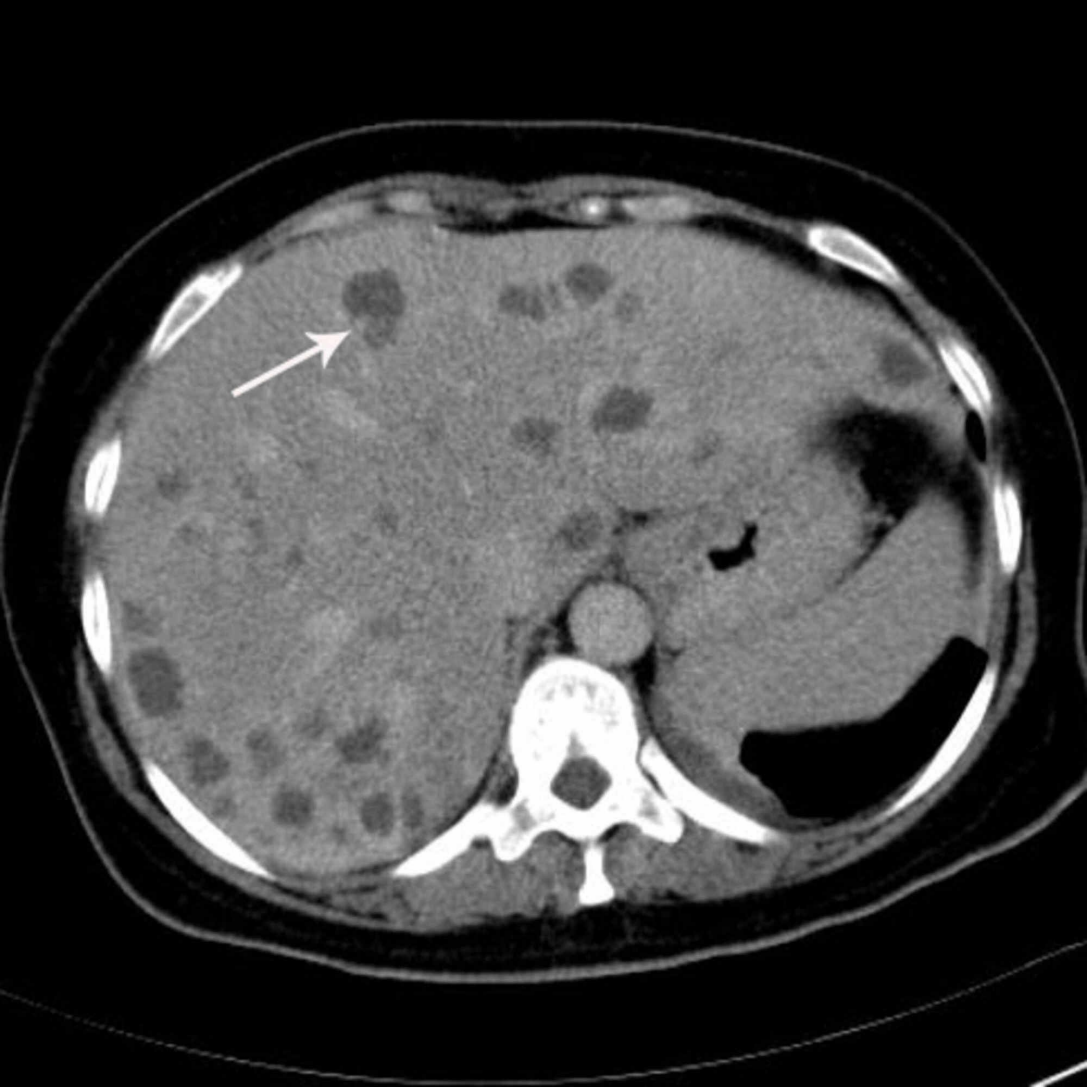
The lesions show peripheral heterogeneous enhancement on delayed venous phase.

**Figure 5:**
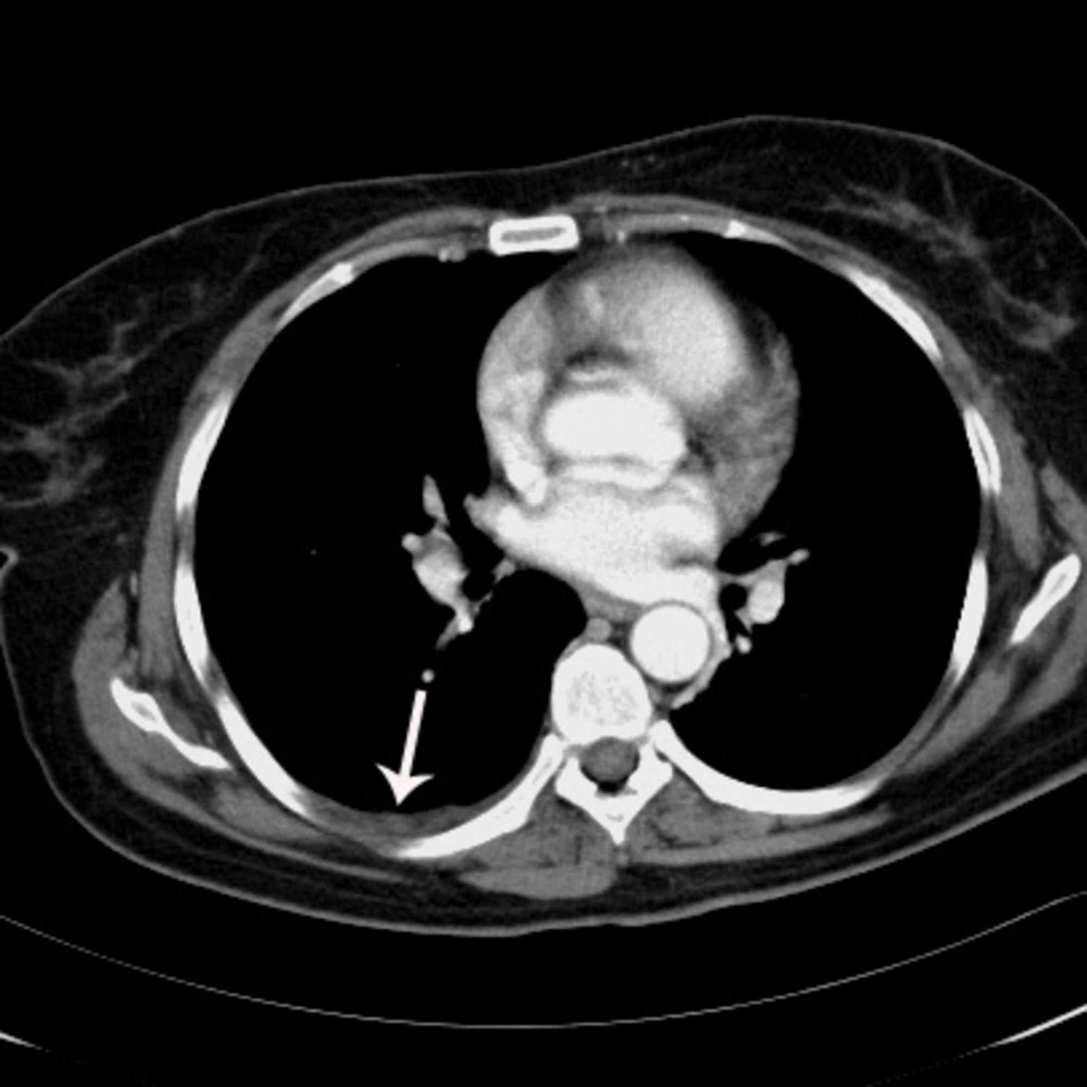
Thoracic MDCT image indicates minimal pleural effusion and pleural thickening with minimally enhanced nodularity (white arrow) seen on the right pleural surface.

**Figure 6:**
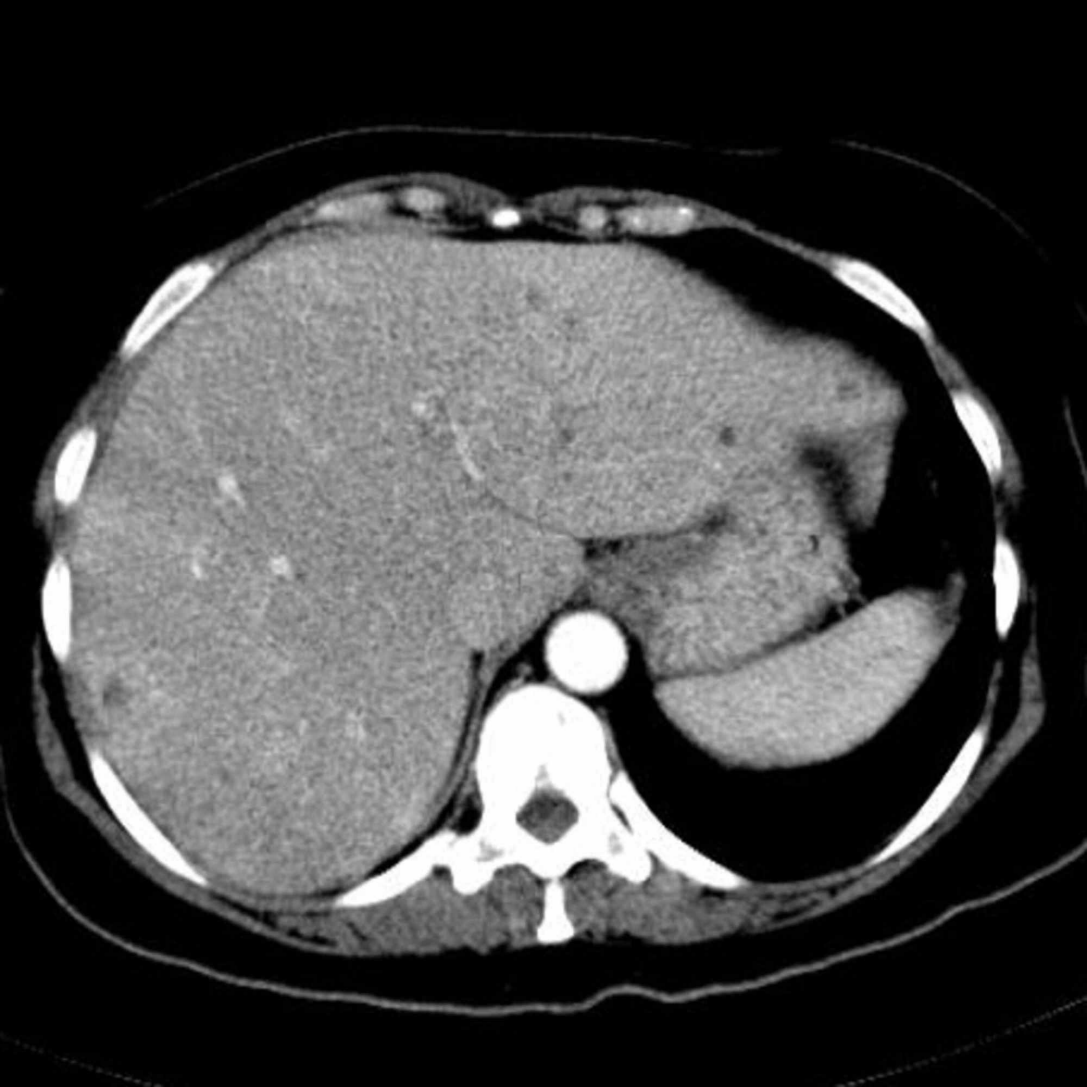
Follow-up contrast-enhanced MDCT image of abdomen demonstrates a tremendous decrease in the size and number of hepatic lesions.

**Figure 7:**
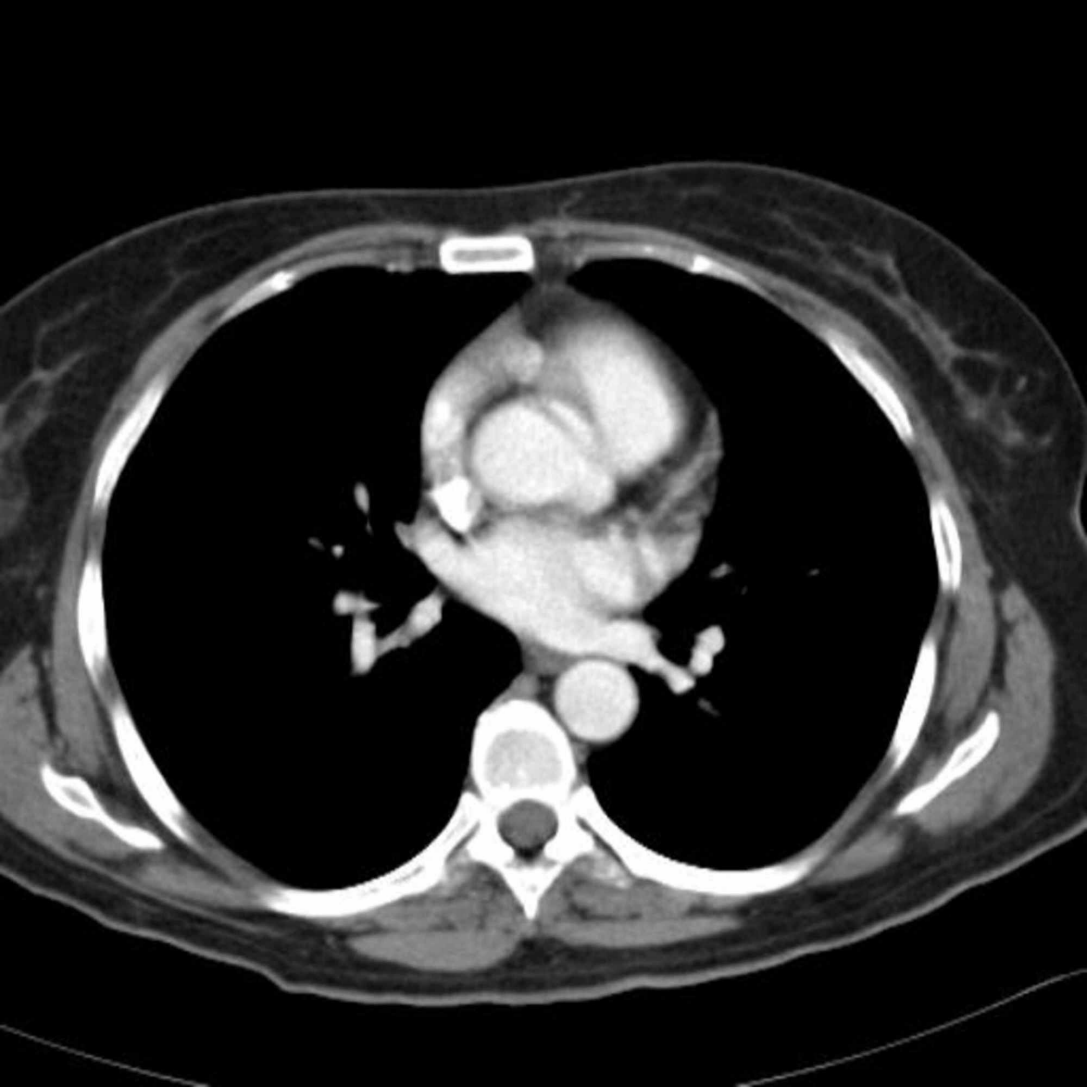
Follow-up contrast-enhanced MDCT image of the thorax reveals the absence of effusion and nodularity on the right pleural surface.

Abdominal precontrast MDCT demonstrated multiple hypodense lesions scattered throughout the liver parenchyma (Figure 1). The largest lesion measured 2.5 cm. Most of the lesions showed heterogeneous peripheral enhancement on the portal and late venous phase images. Some of the lesions also showed contrast enhancement (Figures 2-4). The lesions did not show any clustering. They were thought to be metastatic lesions. There also was a right pleural thickening that showed some nodularity, which were thought to be metastatic lesions (Figure 5). On abdominal and thoracic MDCT examinations, no primary focus was detected. Methicillin-susceptible Staphylococcus aureus and Peptostreptococcus spp. were isolated in the blood cultures. Because both ultrasound and MDCT findings were consistent with metastatic liver disease, ultrasound-guided fine-needle aspiration biopsy was performed from some of the lesions. Hepatic lesions were found to be purulent on aspiration. The pathologic and microbiologic results were compatible with multiple hepatic abscesses. The patient was treated with antibiotic therapy, and within 3-4 weeks, she became afebrile; leukocytosis and elevated C-reactive protein levels returned to normal. On follow-up MDCT two weeks later, the size and number of hepatic lesions had tremendously decreased (Figure 6), and the pleural thickening with nodularity seen on the right pleural surface had disappeared (Figure 7).

## Discussion

Liver abscesses are divided into two main subgroups: pyogenic and nonpyogenic abscesses. Eight to 20 cases per 100000 patients admitted to hospitals have pyogenic liver abscesses [4,5]. These are generally located in the right hepatic lobe (75%). Left hepatic lobe (20%) and caudate lobe (5%) involvement are reported less frequently. The average age of patients that this disease affects is 50-60 years [4].

The most common pathogens causing liver abscesses are Escherichia coli and Klebsiella pneumoniae. Staphylococcus aureus can rarely be isolated, in 20% of the cases or fewer, and it is especially found in childhood [4,5]. Peptostreptococcus spp. have been identified in the range of 3.4-15.5% in the studies [5,6]. Polymicrobial infections have been reported in 7.6-79% of cases [5-7]. Probable causes for the development of hepatic abscesses are: biliary, due to ascending cholangitis; portal vein, due to bacteremia of abdominal sepsis; arterial, due to septicemia; trauma; and local, due to suppuration of adjacent tissues [1-4,8,9]. In nearly 50% of patients, cryptogenic abscesses are seen, and the predisposing factors are generally diabetes mellitus, cirrhosis, malignancy, cardiopulmonary disease, and chronic granulomatous diseases [4,8,9]. Our patient was not on hemodialysis and had no chronic illness other than diabetes, which is a predisposing factor.

The classic triad of the hepatic abscess is right upper quadrant pain, fever, and jaundice, and it is seen only in 10% of patients [2,4,8]. Other clinical presentations are malaise, vomiting, anorexia, fatigue, and weight loss, and none of them is specific [2,4,8]. On physical examination, hepatomegaly, jaundice, and right upper quadrant tenderness may be present [2,4]. Leukocytosis and elevated liver enzymes are found on laboratory examination [2,4,8]. In 50% of patients, the results of blood cultures are positive [8]. The only positive clinical signs in our patient were mild fever and leukocytosis.

On plain chest radiographs, nearly half of all patients may show elevated right diaphragm, atelectasis of the lung bases, and right pleural effusion [3,8]. Ultrasound can be performed, and hepatic abscesses are generally seen as hypoechoic-anechoic cystic lesions, but since they are not always cystic, they may be misdiagnosed sonographically [3].

CT is the most accurate imaging technique for detecting hepatic abscess. On contrast-enhanced hepatic MDCT, abscesses are seen as hypodense masses with an enhanced peripheral rim or capsule. The target appearance of the rim sign is the characteristic sign of the liver abscess [1]. Pyogenic liver abscesses present mostly as single, non-loculated, hypodense lesions, which are usually more than 3 cm in diameter [10]. Multiple small, scattered low-density lesions are seen mostly in Candida abscesses in immunocompromised patients.

Jeffrey and coworkers compared CT findings of 36 patients that had pyogenic liver abscesses with CT findings of 50 patients with hepatic metastases [11]. They identified multiple, small, pyogenic abscesses with a “cluster sign,” which suggests the beginning of coalescence into a single larger abscess cavity, probably showing the early stage of evolution. The cluster sign was seen in five of their patients with pyogenic liver abscess and in one patient with hepatic metastases. They reported that the presence of a cluster sign on CT suggests a pyogenic liver abscess rather than metastases [11]. Hypervascular malignant tumors with central necrosis and metastatic liver lesions also show peripheral enhancement, so that distinguishing these entities from the liver abscess may be difficult, and CT findings of liver abscess and metastatic lesions may be similar. For diagnosis in these cases, pathologic and microbiologic correlation with biopsy is necessary [10]. In our patient, the lesions were similar in size, scattered throughout the liver parenchyma, and some of the lesions had enhancement within the lesion. There were no cluster signs detected in our patient. All these MDCT findings were more consistent with metastatic disease than with a pyogenic abscess.

Antimicrobial therapy combined with percutaneous drainage is the major treatment modality and have 69%-90% cure rates [2,8]. Treatment duration is about 4-6 weeks. Patients having more than two weeks of fever despite percutaneous drainage and appropriate antibiotic therapy undergo surgery [8].

## Conclusions

MDCT with contrast is the radiologic method of choice for detecting liver abscesses. Hepatic abscesses have well-defined CT findings, as discussed above. They may mimic metastatic or necrotic liver tumors with peripheral and lesion enhancement, as seen in abscesses in our case. In patients with delayed diagnosis, hepatic abscess may be fatal. Early diagnosis is crucial to decrease mortality and morbidity rates. In some patients like ours, it may be difficult with MDCT findings to distinguish the abscesses from metastatic lesions. Therefore, microbiologic and pathologic correlation with follow-up may be necessary for these patients.
